# Transfer of the Neurotoxin β-*N*-methylamino-l-alanine (BMAA) in the Agro–Aqua Cycle

**DOI:** 10.3390/md18050244

**Published:** 2020-05-06

**Authors:** Sea-Yong Kim, Sara Rydberg

**Affiliations:** Department of Ecology, Environment and Plant Sciences, Stockholm University, 10654 Stockholm, Sweden; seayong.kim@su.se

**Keywords:** β-*N*-methylamino-l-alanine, Baltic Sea, mussel, chicken, bioaccumulation, agro–aqua cycle

## Abstract

The neurotoxic non-protein amino acid β-*N*-methylamino-l-alanine (BMAA) is connected to the development of neurodegenerative diseases. BMAA has been shown to accumulate in aquatic ecosystems, and filter-feeding molluscs seem particularly susceptible to BMAA accumulation. The blue mussels farmed along the Swedish coastline in the Baltic Sea are, due to their small size, exclusively used to produce feed for chicken and fish in the agro–aqua cycle. We have investigated the possible biotransfer of BMAA from mussels, via mussel-based feed, into chickens. Chickens were divided into two groups, the control and the treatment. BMAA was extracted from the muscle, liver, brain, and eye tissues in both chicken groups; a UPLC-MS/MS method was subsequently used to quantify BMAA. The results indicate detectable concentrations of BMAA in both chicken groups. However, the BMAA concentration in chicken was 5.65 times higher in the treatment group than the control group, with the highest concentration found in muscle tissue extracted from the treatment group chickens. These data suggest that there is a BMAA transfer route within the agro-aqua cycle, so further investigation is recommended before using mussel-based feed in the chicken industry.

## 1. Introduction

β-*N*-methylamino-l-alanine (BMAA) is a neurotoxic non-proteinogenic amino acid naturally produced by a range of ecologically diverse phytoplankton groups such as cyanobacteria, diatoms, and dinoflagellates [1,2,3]. BMAA has been connected to neurodegenerative diseases such as amyotrophic lateral sclerosis (ALS), Alzheimer’s disease, and Parkinson’s disease [1,4]. The compound has been shown to elicit neurotoxicity via various mechanisms, for example, by inducing excitotoxicity as well as erroneous incorporation into proteins, causing protein truncation and aggregation [5,6,7,8,9,10,11,12,13].

Attention to BMAA, as an environmental neurotoxic agent, commenced in the 1940s with the discovery of an unexpectedly high frequency of ALS/PDC among the indigenous Chamorro population of Guam [14,15,16]. The link between BMAA and neurodegenerative disease was first postulated in the 1960s and 1980s, and bioaccumulation and biomagnification of BMAA within the terrestrial food web of Guam were demonstrated, and further evidence was solidified in the early 2000s [1,17,18,19,20,21,22,23]. BMAA has since been demonstrated to bioaccumulate not only in terrestrial ecosystems but also in various aquatic ecosystems, ranging from the subtropical marine aquatic ecosystems of Florida, USA, to the temperate brackish ecosystem of the Baltic Sea, and from the limnic aquatic ecosystems of the subtropical Lake Taihu, China, to the temperate Lake Finjasjön in Sweden [24,25,26]. BMAA bioaccumulation in aquatic ecosystems displays similar patterns, with the highest BMAA concentrations found in bottom-dwelling fish species and filter-feeding aquatic animals such as mussels and oysters [27]. Not only BMAA, but also various other phytoplankton-produced toxins, such as microcystins, cylindrospermopsin, nodularin, and anatoxin-a, have been shown to accumulate in filter-feeding organisms [28,29,30,31]. 

Although the development of neurodegenerative diseases such as ALS and Parkinson’s disease is difficult to directly associate with BMAA exposure via contaminated aquatic food, epidemiological studies show an increased risk of algal toxin exposure not only through aquatic food production but also via other sources such as recreation and aerosolization [32]. For instance, high incidences of ALS were found and reported near several US lakes subjected to high frequencies of algal blooms (e.g., Lake Mascoma, NH, USA and Lake Michigan, WI, USA) [33,34,35], suggesting a relationship between BMAA exposure via aquatic food and neurodegeneration. The same increased incidence of ALS has also been reported from the vicinity of Thau Lagoon, France [36].

The use of filter-feeding organisms (e.g., blue mussels) is a growing industry in the Baltic Sea region and is considered one way to efficiently mitigate eutrophication and clarify the water [37,38,39]. However, the efficiency with which mussel farming reduces eutrophication in the Baltic Sea region has been controversial and questioned because the low salinity of the Baltic Sea causes Baltic Sea blue mussels to be small in size [40,41]. Salinity plays an important role in the morphology and physiology of blue mussels, and in regions where low salinity prevails, such as the Baltic Sea region, the growth rate, maximum size, shell formation, and proportion of meat are negatively affected [40,42]. Consequently, the Baltic Sea blue mussels *Mytilus edulis* and *M. trossulus* are today exclusively used as organic fertilizers and feed for chicken and fish in the so-called agro–aqua cycle [43]. Previously, BMAA transfer routes have been restricted to either terrestrial or aquatic ecosystems [1,27]. However, the use of mussels as chicken feed connects the two ecosystems, and the question of this study is whether or not BMAA accumulates in chicken when the feed is mixed with mussel meat. 

In the study, two groups of chickens were fed, for 36 days, either standard feed spiked with 10% Baltic Sea blue mussel (i.e., *M. edulis* and *trossulus*) (treatment group, *n* = 17) or standard feed (control group, *n* = 12). The tissues investigated were muscle, liver, brain, and eye tissues. The amounts of BMAA in the standard feed and the feed mixed/spiked with mussel meat were also investigated. The data show that the consumption of feed mixed with mussel meat might establish the potential for BMAA transfer within the agro-aqua cycle. In addition, the occurrence of bioaccumulated BMAA found in the eye and liver tissue of the control group chickens fed only standard feed might imply that the eye and liver is more prone to bind BMAA.

## 2. Results

**Figure 1:**
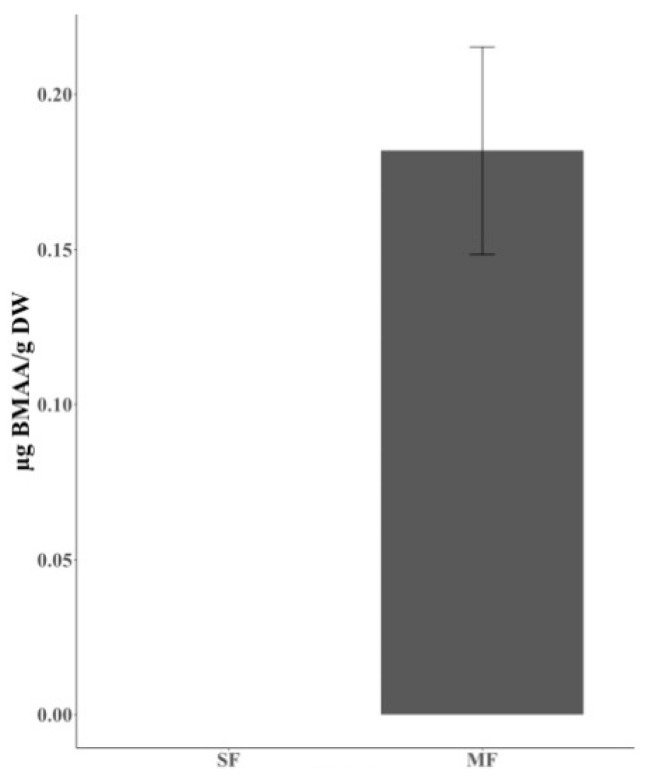
The concentration of BMAA in SF and MF. SF is composed of wheat, barely, oat and maize, and MF consists of 88% of SF and 12% of mussel meat. BMAA concentrations are expressed as µg BMAA/g DW ± SE (n_MF_ = 11). SF, standard feed; MF, mixed fodder with standard feed and mussel meat.

The treatment group (henceforth, TG) displayed a higher growth rate, and on average, the slaughter weight was approximately 50 g higher than in the control group (henceforth, CG) [44]. No abnormal behavior could be observed among the chickens in either the CG or TG. BMAA was quantified using UPLC-MS/MS and expressed in µg BMAA g^−1^ dry weight (DW). The concentration of BMAA extracted from the feed mixed with mussel meat (henceforth, MF) was 0.1818 ± 0.0319 µg BMAA g^−1^ DW (Figure 1, Table 1). No BMAA was detected in the standard feed (henceforth, SF). The BMAA isomers 2,4-diaminobutyric acid (DAB) and N-(2-aminoethyl) glycine (AEG) were detected in samples in both groups along with BMAA (data not shown), however, only BMAA was quantified in the study. 

### 2.1. Total BMAA Concentration in Individual Chickens

BMAA was extracted from four different tissues (i.e., breast muscle, liver, brain, and eye) in both the TG and CG; it was subsequently quantified using UPLC-MS/MS and expressed in µg BMAA g^−1^ DW.

**Figure 2:**
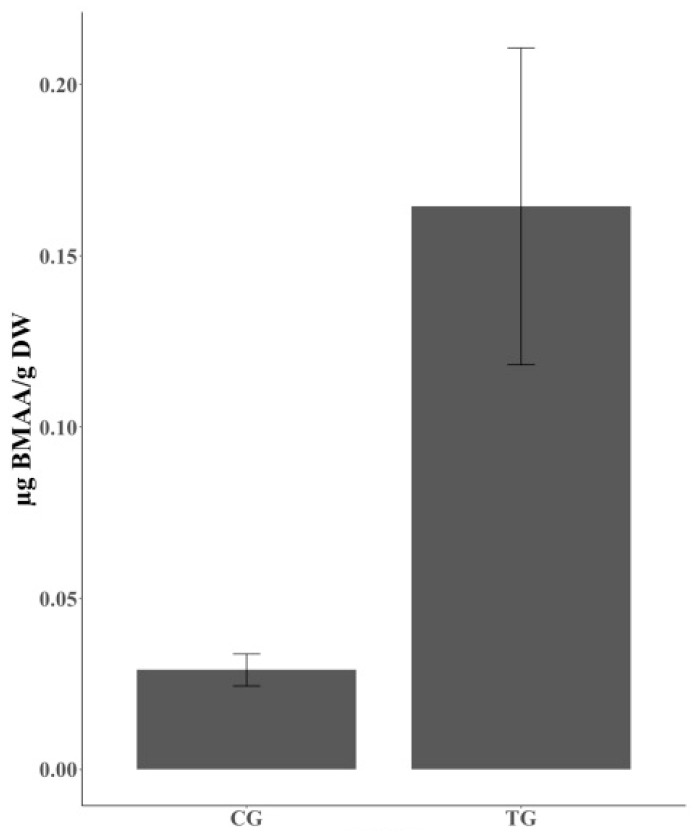
The concentration of BMAA in individual chicken belonging to CG and TG. CG fed on standard feed (SF), and TG on the mixed feed with standard feed and mussel meat (MF) for 36 days. BMAA concentrations are expressed as µg BMAA/g DW ± SE (n_CG_ = 8, n_TG_ = 12). CG, control group; TG, treatment group.

BMAA was unexpectedly detected in individual CG chickens, though there was a clear gap in the amount of accumulated BMAA between the chickens in the CG and TG (Figure 2). In the TG, BMAA could be detected in 12 of 17 individual chickens and the average concentration of total BMAA was 0.1644 ± 0.0443 µg BMAA g^−1^ DW (Figure 2, Table 2). BMAA could also be detected in 8 of 12 individual chickens in the CG and the average concentration of total BMAA was 0.0291 ± 0.0044 µg BMAA g^−1^ DW. This concentration is approximately 5.65 times lower than the concentration of BMAA found in the TG (Figure 2, Table 2).

### 2.2. BMAA Concentration in Protein-Associated and Free Forms

**Figure 3:**
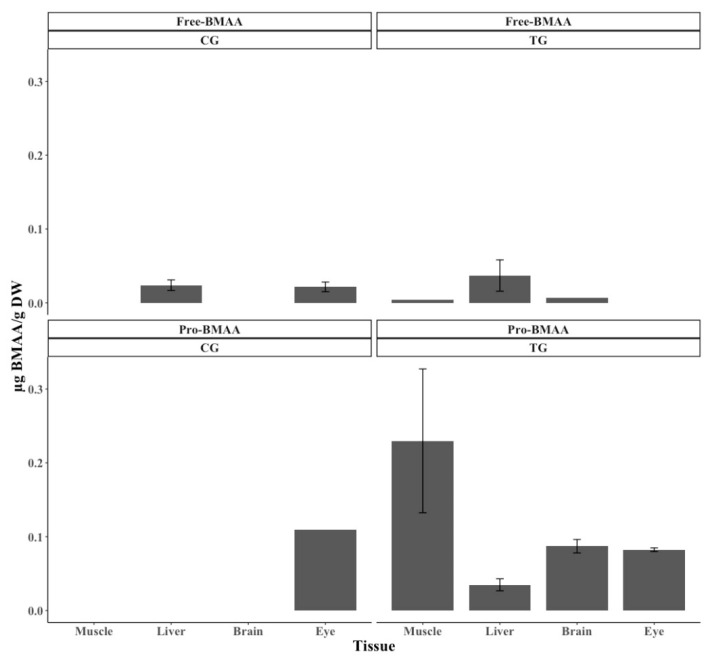
The concentration of Free- and Pro-BMAA from each tissue in CG and TG. Free-BMAA is detected from liver and eye of CG, and from muscle, liver, brain of TG. Pro-BMAA is detected from eye of CG, and from all tissues of TG. BMAA concentrations are expressed as µg BMAA/g DW ± SE (Free-BMAA, n_CG-Liver_ = 7, n_CG-Eye_ = 3, n_TG-Muscle_ =1, n_TG-Liver_ = 3, n_TG-Brain_ = 1; Pro-BMAA, n_CG-Eye_ = 1, n_TG-Muscle_ = 4, n_TG-Liver_ = 2, n_TG-Brain_ = 8, n_TG-Eye_ = 2). Free-BMAA, free form of BMAA; Pro-BMAA, protein-associated form of BMAA; CG, control group; TG, treatment group.

BMAA in the protein-associated form is the main cause of the large difference in the total amount of BMAA accumulated within the individual chickens belonging to the TG versus CG (Figure 2 and Figure 3, Table 2 and Table 3). The highest and lowest BMAA concentrations in protein-associated form were 0.230 ± 0.0974 µg BMAA g^−1^ DW from muscle tissue and 0.0348 ± 0.0081 µg BMAA g^−1^ DW from liver tissue in TG chickens, respectively (Figure 3, Table 3). In terms of free BMAA, the highest concentration was 0.0370 ± 0.0212 µg BMAA g^−1^ DW found in liver tissue of TG chickens and the lowest was 0.0045 µg BMAA g^−1^ DW in muscle tissue of TG chickens (Figure 3, Table 3).

BMAA was widely distributed and detected in all four tissue types of TG chickens, while BMAA could only be detected in liver and eye tissues in CG chickens (Figure 3, Table 3). In addition, the type of BMAA (i.e., protein-associated and free forms) in the two groups (i.e., TG and CG) affected the pattern of BMAA accumulation. Protein-associated BMAA in the TG primarily accumulated in muscle tissue, where the highest concentrations could be found, while the lowest concentration of BMAA was found in liver tissue (Figure 3, Table 3). The numbers of TG chickens containing protein-associated BMAA in muscle, liver, brain, and eye tissues were 4/17, 2/17, 8/17, and 2/17, and the average concentrations were 0.230 ± 0.0974, 0.0348 ± 0.0081, 0.0871 ± 0.0091, and 0.0823 ± 0.0025 µg BMAA g^−1^ DW, respectively. The numbers of TG chickens containing free BMAA in muscle, liver, and brain tissues were 1/17, 3/17, and 1/17, and the average concentrations were 0.0045, 0.0370 ± 0.0212, and 0.0072 µg BMAA g^−1^ DW, respectively. No free form BMAA could be detected in eye tissue of the TG chickens. In contrast, the highest concentrations of protein-associated and free BMAA in the CG chickens were detected in eye and liver tissues. The number of CG chickens containing protein-associated BMAA in eye tissue was 1/17, with an average concentration of 0.109 µg BMAA g^−1^ DW. The numbers of CG chickens containing free BMAA in liver and eye tissues were 7/12 and 3/12 with average concentrations of 0.0240 ± 0.0071 and 0.0216 ± 0.0065 µg BMAA g^−1^ DW, respectively.

## 3. Discussion

In this study, we can conclude that the biomagnification of BMAA is not constrained to closed ecosystems, but can expand from aquatic to agricultural ecosystems, connecting the two. This conclusion is based on the finding of accumulated and biomagnified BMAA in chickens fed standard feed spiked with mussel meat, which contains BMAA. The concentrations of BMAA found in the mussel *M. edulis* farmed on the Swedish west coast have earlier been reported to range between 0.151 ± 0.009 and 0.201 ± 0.07 µg BMAA g^−1^ DW [27] (Table 4). In contrast, other bioaccumulation studies of bivalve molluscs, including mussels and oysters, have found large variation in BMAA concentration depending on species and origin: 0.6–9.7 µg BMAA g^−1^ DW in Thau Lagoon, France [36,45], and 6.8–46.9 µg BMAA g^−1^ DW in Louisiana and Mississippi, USA [46] (Table 4). The amount of BMAA found in the MF used in this study was 0.1818 ± 0.0319 µg BMAA g^−1^ DW. Although the SF was spiked with only 10% mussel meat, the concentrations of detected BMAA were similar to those previously found by Jonasson et al. [27]. The ability of mussels to accumulate and remove BMAA from surrounding media was earlier demonstrated by Downing et al. in 2014 [47]. In that study, four species of freshwater mussel, i.e., *Anodonta cygnea*, *Unio tumidus*, *Dreissena polymorpha*, and *Corbicula javanicus*, were artificially supplemented with isotopically labelled BMAA. All mussels could take up BMAA from the media, and 45%–98% of the exogenously applied BMAA was removed from the media after 48 h; after 24 h, the concentrations of BMAA in the mussels were 0.4–12.5 µg BMAA number^−1^ mussel [48].

In the current study, the average concentration of BMAA detected in individual TG chickens was 0.1644 ± 0.0443 µg BMAA g^−1^ DW (Figure 2, Table 2). Given the short growing period of 36 days and the low percentage of mussel meat mixed into the SF, the accumulated and biomagnified concentrations of BMAA found in TG chickens are severe and cannot be ignored. The BMAA concentrations could be expected to become even higher if the chickens were to grow for more than 36 days or if more mussel meat were mixed into the SF.

The reported concentrations of BMAA accumulated in the chickens vary greatly, and such large variation in bioaccumulated and biomagnified BMAA concentrations has been seen in other studies as well. For instance, the concentrations of BMAA accumulated in the flying foxes *Pteropus mariannus* and *P. yapensis* collected in Guam ranged from 13 to 1859 µg BMAA g^−1^ DW [49]. Large variation in detected BMAA concentrations has also been confirmed in various fish species in, for example, limnic ecosystems: *Gymnocephalus cernua* (0.00320 ± 0.00329 to 0.00864 ± 0.00479 µg BMAA g^−1^ DW), *Tinca tinca* (0.00141 to 0.00561 µg BMAA g^−1^ DW), and *Abramis brama* (0.00103 ± 0.00027 to 0.00200 ± 0.00173 µg BMAA g^−1^ DW) [26], and in the Baltic Sea: *Osmerus eperlanus* (0.016 ± 0.0009 to 0.24 ± 0.003 µg BMAA g^−1^ DW), *Scophthalmus maximus* (0.0008 ± 0.0003 to 1.29 ± 0.03 µg BMAA g^−1^ DW), *Clupea harengus* (0.0007 ± 0.00008 to 0.010 ± 0.001 µg BMAA g^−1^ DW), and *Coregonus lavaretus* (0.0019 ± 0.00007 to 0.059 ± 0.004 µg BMAA g^−1^ DW) [27]. The concentration of BMAA found in the TG chickens ranged from 0 to 0.0370 ± 0.0212 µg BMAA g^−1^ DW (free form) and from 0.0348 ± 0.0081 to 0.230 ± 0.0974 µg BMAA g^−1^ DW (protein-associated form) (Figure 2, Table 2). There might be several explanations for these large variations, such as biological and methodological factors as well as exposure duration. For instance, as a biological reason, accumulation of BMAA in tissues is likely species-dependent and, coincidentally, as a methodological reason, different studies use different extraction methods and analytical methodologies to quantify BMAA: for example, HPLC-FLD was used by Banack et al. [49] and UPLC-MSMS was used by Jonasson et al. [27], Lage et al. [26], and the present authors. The actual duration of exposure to BMAA cannot be verified in the studies of Banack et al. [49] and Jonasson et al. [27], whereas the exposure duration in the present study was 36 days.

The distribution of accumulated BMAA in vertebrates varies according to species and their environments. In this study, the highest BMAA concentration was detected in muscle tissue, the tissue mostly consumed by humans. The second highest concentration was confirmed in brain, followed by eye and liver tissues (Figure 3, Table 3). The English poultry breed, famous for high egg and meat production (especially in breast muscle tissue) was used during the experiment. The high protein production in the muscle tissue, compared to the other tissues, might cause the detected high level of bioaccumulated protein-associated BMAA in the chicken muscle. It has earlier been suggested that BMAA can be misincorporated in place of L-serine in human proteins [13]. In the study of Dunlop et al. (2013), incorporation of BMAA into proteins was dependent on protein synthesis [13]. Banack et al. [49] found that the hair of *P. mariannus mariannus* contained the highest concentration of BMAA, followed by muscle, liver, skin, and kidney tissues, while in most fish samples collected from the Baltic Sea, the highest BMAA concentrations were detected in brain tissue followed by muscle tissue [27]. In contrast to the results reported in the Baltic Sea study [27], the highest concentrations of BMAA detected in fish collected in the limnic ecosystem of Lake Finjasjön varied depending on fish species [26]. All these data suggest that the distribution pattern of BMAA accumulation within vertebrates is related to various factors such as species, diet, and ecological niche.

Another aspect of this study is the unexpectedly large number (8/12) of individual chickens fed only SF that contained BMAA (Table 2). Seven of 12 and 3 of 12 individuals in the CG contained detectable and quantifiable concentrations, respectively, of free BMAA in both liver and eye tissues (Figure 3, Table 3). One of 12 of these individual chickens also contained protein-associated BMAA in eye tissue. The CG chickens may be exposed to BMAA via either water or the previous generation [50]. Considering the number of individual CG chickens containing BMAA, our results suggest that eye and liver might be the most sensitive tissues in chickens in terms of BMAA accumulation. One interesting point is that BMAA was accumulated in eye tissue in both free and protein-associated forms.

Notably, 3H-BMAA was shown to preferentially accumulate in the pigmented parts of melanin- and neuromelanin-containing tissues such as brain, eye, hair, liver, and the melanocytes surrounding blood vessels and visceral organs in mice and frogs, further suggesting that these are possible BMAA reservoirs [51]. Throughout Karlsson et al.’s [51] experimental period, the localization and concentration of 3H-BMAA were maintained in mouse eye tissue, which does not contain neuromelanin, and in frog brain tissue, which does contain neuromelanin; no BMAA was detected in the eye tissue of adult albino mice [51]. However, further studies need to be performed in order to prove the theory of eye as a biomarker for BMAA bioaccumulation where the dose- and time-dependency of BMAA bioaccumulation is investigated as well. The eye has been suggested to be a possible biomarker of neurodegenerative diseases such as Alzheimer’s disease, Parkinson’s disease, and ALS/PDC. More than 50% of ALS/PDC patients in Guam were diagnosed with an uncommon retinal disease—a pigmentary retinopathy [52,53]—and abnormalities of the vision function have been reported in advanced-stage Alzheimer’s disease [54,55]. In addition, numerous studies have recently discovered other eye deficiencies and symptoms accompanying neurodegeneration [56,57,58].

The present data clearly demonstrate the risk of using the blue mussels *M. edulis* and *trossulus* as ingredients in the industrial production of animal feed. The trial data show that BMAA bioaccumulated in all analyzed tissues dissected from the individual chickens. Furthermore, the highest amount of bioaccumulated BMAA was found in the muscle tissue of chickens fed mussel meal. Humans commonly consume the muscle tissue of chickens, so this might lead to a higher risk of neurotoxin exposure if the chicken feed is mixed with mussels.

## 4. Materials and Methods

### 4.1. The Culture and Collection of the Chicken Sample

Two groups of chickens grew for 36 days, from 21 May to 25 June 2018. The experiment was conducted in collaboration with the Kalmar Sound Commission and the company Ölands Chicken AB (Mörbylånga, Sweden), and was performed under controlled conditions, with the chickens being monitored by a bird specialist veterinarian. Twenty-four hours after hatching, the TG (*n* = 17) was fed MF comprising 90% SF and 10% Baltic Sea blue mussels *M. edulis* and *M. trossulus*, while the CG (*n* = 12) was fed 100% SF comprising wheat, barley, oats, and maize. The mussels *M. edulis* and *M. trossulus*, used in producing the MF, were collected from the mussel farm located at Hasselö in Västervik municipality (Baltic Sea). Four types of tissues, i.e., muscle, liver, brain, and eye, were dissected by the veterinarian. The dissected samples were directly stored in liquid nitrogen while being transferred to the laboratory. Chicken specimens were stored at −80 °C until BMAA was extracted.

### 4.2. Sample Preparation and BMAA Extraction

The samples of the different chicken tissues (i.e., muscle, liver, brain, and eye) as well as the different feeds (i.e., SF and MF) were lyophilized using a CoolSafe freeze-dryer (SCANVAC, Stockholm, Sweden) at −110 °C. The numbers of biological replicates were as follows: TG, *n* = 17; CG, *n* = 12; MF, *n* = 12; and SF, *n* = 12. Samples were ground and once again lyophilized using a CoolSafe freeze-dryer. On average, 30 mg DW of ground tissue was used for extracting BMAA. The BMAA extraction procedure was based on that of Murch et al. [23] with minor alterations [36,59]. Samples were dissolved in 1 mL of 0.1 mol L^−1^ TCA before lyophilization by means of sonication (Sonopuls Model HD 2070; Bandelin Electronic, Berlin, Germany) for 3 min at 70% efficiency. The procedure was performed in an ice-water bath to prevent protein degradation. Samples were homogenized by vortexing and the proteins were incubated for 48 h at 4 °C for protein precipitation. To separate the precipitated protein pellet containing the protein-associated BMAA and the supernatant containing the free BMAA, samples were centrifuged for 10 min at 10,000× *g* at 4 °C. To further ensure efficient protein precipitation and separation between the free and protein-associated forms of BMAA, yet another 500 µL of 0.1 M TCA was added to the pellet and the samples were once again incubated for 1 h at 4 °C. Subsequently, samples were centrifuged at 10,000× *g* and 4 °C for 10 min. Both the supernatant (from the first and second rounds of centrifugation) and the pellets containing the free and protein-associated forms of BMAA, respectively, were lyophilized in a freeze dryer, and hydrolyzed in 600 µL of 6 M HCl for 20 h at 110 °C. Both fractions were filtered with a centrifugal filter unit (Ultrafree-MC centrifugal filter; Merck Millipore, Billerica, MA, USA) for 1 min at 10,000× *g*. Samples were frozen at −80 °C for lyophilization. The lyophilized samples were later diluted before UPLC-MS/MS analysis. Samples of free BMAA were diluted with 2 mL of 20 mM HCl. Samples of protein-associated BMAA were diluted with 20 mM HCl to obtain an optimum ratio of protein to derivatization agent [59].

### 4.3. Protein Extraction and Quantification

Aliquots (10 µL) of the TCA-precipitated protein sample taken after adding TCA in the second round were used to measure the total protein contents. Three technical replicates of each biological replicate were used to determine the total amount of protein per biological replicate. The amount was quantified using the BioRad RC/DC kit (Bio-Rad, Sundbyberg, Sweden) according to the manufacturer’s instructions. Spectrophotometric measurements were made using a µQuant monochromatic microplate spectrophotometer (BioTek, Winooski, VT, USA).

### 4.4. Ultra-Performance Liquid Chromatography–Tandem Mass Spectrometry

The diluted samples were derivatized with AccQ-Tag using a WAT052880 AccQ-Tag kit (Waters, Milford, MA, USA), i.e., 70 µL of borate buffer and 30 µL of AQC. An Acquity UPLC system coupled to a Xevo-TQ-MS system (Waters) was used for BMAA analysis, based on the Lage et al. [58] method with minor alterations. Separation was conducted using an AccQ-Tag Ultra C18 column (100 × 2.1 mm, 1.7 μm particle size; Waters) and a binary mobile phase (eluent A: 0.01% formic acid in water; eluent B: 0.01% formic acid in methanol) delivered at a flow rate of 0.5 mL min^−1^. The linear gradient elution program was as follows: 1% B for 0–0.5 min, 60% B for 4 min, 99% B for 4.10 min, and 1% B for 5.20–6.20 min; injection volumes were 20 μL. Analytes were analyzed in positive ion mode using electrospray ionization and selected reaction monitoring (SRM) scan mode using the following transitions: general to all three analytes, 459.1 > 119.08; 4-diaminobutyric acid (DAB) diagnostic fragment, 459.1 > 188.1; N-(2-aminoethyl) glycine (AEG) diagnostic fragment, 459.1 > 214.1; and BMAA diagnostic fragment, 459.1 > 258.09. The instrument parameters were as follows: cone voltage, 30 V; source temperature, 150 °C; desolvation temperature, 550 °C; cone gas flow, 20 L h^−1^; desolvation gas flow, 1000 L h^−1^; collision gas flow, 0.15 mL min^−1^; and collision energy, 26 V. MassLynx V4.1 software (Waters, Stockholm, Sweden) was used to analyze the acquired chromatographic data.

### 4.5. Standard Curve for BMAA Quantification

Samples with no detected BMAA were selected for constructing a standard curve in order to quantify BMAA. Calibration curves were made for each tissue type (i.e., muscle, liver, brain, and eye) and BMAA type (i.e., free and protein-associated forms) at six concentrations for the free form (i.e., 0.3, 1.2, 3, 6, 12, and 30 ng mL^−1^) and seven for the protein-associated form (i.e., 0.3, 1.2, 3, 6, 12, 30, and 60 ng mL^−1^). Different tissue and BMAA types displayed different limits of quantification (LOQ). Concentrations below 0.3 (for muscle, liver, and eye tissues for the protein-associated form and for muscle, liver, and brain tissues for the free form) or 1.2 (for brain tissues for the protein-associated form and for eye tissues for the free form) ng mL^−1^ (0.425 nM) were below the LOQ, so the samples were considered non-quantifiable (NQ). Samples with no detected BMAA peak were considered non-detectable (ND). Most MF samples (11/12) contained BMAA, so to confirm the BMAA concentration, H3-BMAA in two concentrations (0.2 and 0.5 ng mL^−1^), representing the range of BMAA concentrations in the sample, was added to quantify BMAA concentration.

## Figures and Tables

**Table 1:** The concentration of BMAA in SF and MF. BMAA was detected only from 11 out of 12 biological replicates of MF. “Number” indicates the number of biological replicate accumulating BMAA. BMAA concentrations are calculated from 11 out of 12 biological replicates containing BMAA. BMAA concentrations are expressed as µg BMAA/g DW ± SE. SF, standard feed; MF, mixed fodder with standard feed and mussel meat.

Feed	BMAA µg/g DW	
	Number	Concentration
**SF**	0/12	ND
**MF**	11/12	0.1818 ± 0.0319

**Table 2:** The concentration of BMAA in individual CG and TG chicken. BMAA was detected from 8 out of 12 individual chickens in CG, and 12 out of 17 in TG. “Number” indicates the number of biological replicate accumulating BMAA. BMAA concentrations are calculated from the individual chicken containing BMAA and expressed as µg BMAA/g DW ± SE. CG, control group; TG, treatment group.

Chicken Group	BMAA µg/g DW	
	Number	Concentration
**CG**	8/12	0.0291 ± 0.0044
**TG**	12/17	0.1644 ± 0.0443

**Table 3:** The concentration of Free- and Pro-BMAA from each tissue in the CG and TG. BMAA was extracted from four different tissues, muscle, liver, brain and eye, of both chicken groups CG and TG. “Number” indicates the number of biological replicate accumulating BMAA. BMAA concentrations are calculated from the tissue containing BMAA and expressed as µg BMAA/g DW ± SE. Free-BMAA, free form of BMAA; Pro-BMAA, protein-associated form of BMAA; CG, control chicken group; TG, treated chicken group; NQ, non-quantifiable.

Tissue.	BMAA µg/g DW							
	Free-BMAA				Pro-BMAA			
	CG		TG		CG		TG	
	Number	Concentration	Number	Concentration	Number	Concentration	Number	Concentration
**Muscle**	0/12	NQ	1/17	0.0045	0/12	NQ	4/17	0.2300 ± 0.0974
**Liver**	7/12	0.0240 ± 0.0071	3/17	0.0370 ± 0.0212	0/12	NQ	2/17	0.0348 ± 0.0081
**Brain**	0/12	NQ	1/17	0.0072	0/12	NQ	8/17	0.0871 ± 0.0091
**Eye**	3/12	0.0216 ± 0.0065	0/17	NQ	1/12	0.109	2/17	0.0823 ± 0.0025

**Table 4:** The concentration of accumulated BMAA in different organisms.

Common Name (Species)	Concentration (µg BMAA g^−1^ DW)	References
**Mussel (*Mytilus edulis*)**	0.151 ± 0.009 to 0.201 ± 0.07	[27]
**Mussel (*Mytilus galloprovincialis*)**	~9.7	[45]
**Mussel (*Mytilus galloprovincialis*)**	1.8 to 6.0	[36]
**Oyster (*Crassostrea gigas*)**	0.6 ± 0.07 to 1.6 ± 0.82	[36]
**Oyster (*Crassostrea virginica*)**	6.8 to 46.9	[46]
**Flying fox (*Pteropus mariannus* and *P. Yapensis*)**	13 to 1859	[49]
**Fish (*Gymnocephalus cernua*)**	0.00320 ± 0.00329 to 0.00864 ± 0.00479	[26]
**Fish (*Tinca tinca*)**	0.00141 to 0.00561	[26]
**Fish (*Abramis brama*)**	0.00103 ± 0.00027 to 0.00200 ± 0.00173	[26]
**Fish (*Osmerus eperlanus*)**	0.016 ± 0.0009 to 0.24 ± 0.003	[27]
**Fish (*Scophthalmus maximus*)**	0.0008 ± 0.0003 to 1.29 ± 0.03	[27]
**Fish (*Clupea harengus*)**	0.0007 ± 0.00008 to 0.010 ± 0.001	[27]
**Fish (*Coregonus lavaretus*)**	0.0019 ± 7.E-5 to 0.059 ± 0.004	[27]
